# Dopamine modulation of spike-timing-dependent plasticity for spatio-temporal spike pattern detection in single neurons

**DOI:** 10.3389/fncom.2026.1777273

**Published:** 2026-07-31

**Authors:** Ayaka Kotajima, Shunta Furuichi, Takashi Kohno

**Affiliations:** 1 Tokyo Metropolitan Shinjuku Yamabuki Senior High School, Tokyo, Japan; 2 Department of Electrical Engineering and Information Systems, Graduate School of Engineering, The University of Tokyo, Tokyo, Japan; 3 Institute of Industrial Science, The University of Tokyo, Tokyo, Japan

**Keywords:** dopamine, eligibility trance, spike pattern detection, spiking neural network model, STDP

## Abstract

Dopamine (DA) is an important neuromodulator that has been suggested to play key roles in a range of neuropsychiatric disorders. However, its computational impact at a general single-neuron level has not been elucidated. Here, we extended a spike-timing-dependent plasticity (STDP)-based single-neuron spatio-temporal pattern detection model by incorporating a DA input and DA-type STDP modulation. We analyzed the direct effect of the DA-type STDP curve and the effect of DA release. We found that the DA-type STDP curve accelerates learning but may promote coarse potentiation of the synaptic efficacy, which leads to false detections. It was also shown that DA can improve the pattern-detection performance up to a 44% success rate (2.44 × control) with no false detection, but an excessive DA release or a DA release with too short a delay induces false detection. These results suggest that DA can maintain the pattern detection ability only when released within a limited concentration and a sufficient delay.

## 1 Introduction

Dopamine (DA) is one of the major neuromodulators in the central nervous system. By acting on widespread receptors and modulating synaptic plasticity and neuronal excitability, DA influences key cognitive functions such as attention, learning, and decision-making (Bromberg-Martin et al., 2010). Dysregulation of dopaminergic signaling has also been implicated in a range of neuropsychiatric conditions, including attention-deficit/hyperactivity disorder (ADHD), autism spectrum disorder (ASD), and schizophrenia (Cai et al., 2021). Importantly, these disorders place a substantial burden on individuals and society. Approximately 1/8 of the world's population is afflicted (GBD 2019 Mental Disorders Collaborators, 2022), and current treatments still have major limitations. For example, while pharmacological interventions can be effective for some symptom domains, cognitive impairments in schizophrenia remain difficult to treat and continue to be a central obstacle to functional recovery (McCutcheon et al., 2023).

A large body of prior works has investigated how DA relates to brain function and dysfunction at macroscopic and clinical levels [e.g., (McCutcheon et al., 2019; Rogeau et al., 2025; MacDonald et al., 2024; Pavăl, 2017)]. These studies have established important associations between dopaminergic signaling and population-level or systems-level neural activity. However, explaining the mechanistic principles of the DA's effects by such associations remains challenging. Because the brain is an extremely complex dynamical system, a simple one-to-one mapping between stimulus and response does not elucidate the mechanisms of the DA's pharmacological effects at the neural circuit level.

On the other hand, the neural circuit level analyses are still limited. To the best of our knowledge, there is little preceding work other than the study by Wert-Carvajal et al. (2022). They studied an abstracted hippocampal network using a spiking neural network (SNN) model and analyzed the effects of DA- and 5-HT-type spike-timing-dependent plasticity (STDP) curves, demonstrating their impact at the level of the abstracted hippocampal circuit. The effect of DA on more fundamental single neurons' information processing has not yet been analyzed.

In this study, we investigated the effect of DA on a single neuron by extending a spatio-temporal spike pattern detection model (Masquelier et al., 2008) based on STDP (Markram et al., 1997; Bi and Poo, 1998). A DA input was incorporated into the single-neuron model by Masquelier et al. (2008). Here, the effect of DA was modeled by a modified STDP learning curve reported by Zhang et al. (2009) (DA-type STDP curve). Here, we aim to develop a general model for single-neuron information processing rather than a faithful reproduction of any specific anatomical circuit. Because Zhang et al. (2009) was the only study on the DA-type STDP curve we could find, it was incorporated in our model, independent of the precise anatomical source. We evaluated how the DA-type STDP curve modulates the performance of the spike pattern detection task with two setups. The first aimed to evaluate the direct effect of the DA-type STDP curve, and the second aimed to evaluate the effect of DA release under a more biologically realistic condition.

By focusing on a single neuron, we aim to provide a foundation for a mechanistic and bottom-up perspective on the dopaminergic modulation of neural circuits' computation. This work is intended as a first step toward bridging macroscopic clinical observations and circuit-level principles, ultimately contributing to a clearer understanding of how DA can contribute to cortical information processing through synaptic plasticity.

## 2 Methods

### 2.1 Model

We incorporated a DA input into the spatio-temporal spike pattern detection model by Masquelier et al. (2008). The spiking dynamics are composed of a leaky integrate-and-fire (LIF) spiking mechanism with a double-exponential synapse model. In the study by Masquelier et al. (2008), an asymmetric STDP was shown to enable a neuron to detect a spatio-temporal spike pattern embedded in noise (Poisson spikes). We adopted this model among single-neuron learning models because it provides one of the best balances between biological plausibility and computational cost.

The model equations are as follows. Let the membrane potential be


$$\begin{array}{l} v=\eta +\overset{N}{\underset{j=1}{\sum }}w_{j}\varepsilon_{j}, \end{array}$$ (1)

where ε_*j*_ is the excitatory postsynaptic potential (EPSP) raised by the *j*-th synapse, η is a variable generating spiking activity, and *w*_*j*_ is the synaptic weight of the *j*-th synapse. The total number of synaptic inputs *N* is 2,000. Variables η and ε_*j*_ follow


$$\begin{array}{ll} \frac{d\eta }{dt} & =-\frac{\eta }{\tau_{m}}+\theta K_{2}\left(\frac{1}{\tau_{m}}-\frac{1}{\tau_{s}}\right)x_{s}, \end{array}$$ (2)

$$\begin{array}{ll} \frac{d\varepsilon_{j}}{dt} & =-\frac{\varepsilon_{j}}{\tau_{m}}-K\left(\frac{1}{\tau_{m}}-\frac{1}{\tau_{s}}\right)y_{sj}, \end{array}$$ (3)

where θ = 500 is the threshold, τ_*m*_ = 10 ms is the membrane time constant, τ_*s*_ = 2.5 ms is the synaptic time constant, and *K* = 2.12, *K*_2_ = 4 are constants. The variables on the right-hand side of the above equations, *x*_*s*_ and *y*_*sj*_, evolve as


$$\begin{array}{ll} \frac{dx_{s}}{dt} & =-\frac{x_{s}}{\tau_{s}}, \end{array}$$ (4)

$$\begin{array}{ll} \frac{dy_{sj}}{dt} & =-\frac{y_{sj}}{\tau_{s}}. \end{array}$$ (5)

When *v* exceeds the threshold, the variables are reset to η = 2θ, ε_*j*_ = 0, and *x*_*s*_ = 1. At an arrival time *t*_*j*_ of a presynaptic spike on the *j*-th synapse, *y*_*sj*_ is reset to 1.

**Figure 1:**
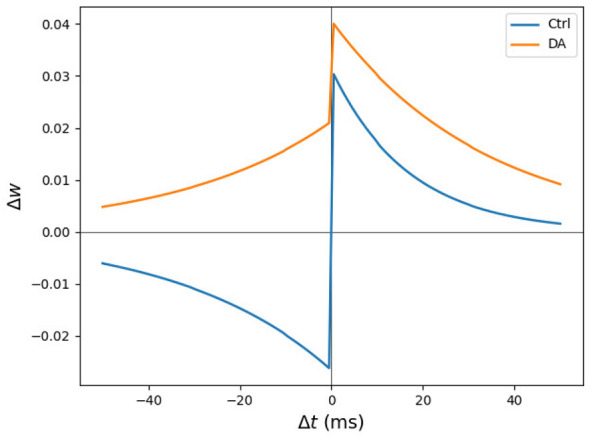
Spike-timing-dependent plasticity (STDP) curves without Dopamine (DA; blue plot) and with DA (orange plot). An asymmetric STDP curve was assumed for the control condition (without DA), while an all-positive curve was assumed under the existence of DA (Zhang et al., 2009).

Synaptic weights *w*_*j*_ are updated by an asymmetric STDP rule. The change Δ*w*_*j*_ of *w*_*j*_ depends on the time difference between the presynaptic spike arrival time at synapse *j*, *t*_pre*j*_, and the neuron's spike time *t*_post_. The baseline function is shown as Ctrl (blue) in Figure 1. Based on the experimental findings of Zhang et al. (2009) on how DA modulates the STDP curve, we modeled the STDP curve under DA (orange).

The STDP curve is


$$\Delta w_{j}\left(t_{j}\right)=\{\begin{array}{cc} a^{+}\exp\left(\frac{\Delta t_{j}}{\tau^{+}}\right), & \Delta t_{j}\leq 0,\\ -a^{-}\exp\left(-\frac{\Delta t_{j}}{\tau^{-}}\right), & \Delta t_{j}>0, \end{array}$$ (6)

where Δ*t*_*j*_ = *t*_post_−*t*_pre*j*_, τ^+^, τ^−^ are time constants, and *a*^+^, *a*^−^ are amplitude constants. For the baseline, we used (τ^+^ = 16.8 ms, τ^−^ = 33.7 ms, *a*^+^ = 0.03125, *a*^−^ = 0.85 × 0.03125) (same as Masquelier et al. (2008)). Under DA, $\left(\tau_{\text{DA}}^{+}=2.0\times 16.8\text{ ms},\;\tau_{\text{DA}}^{-}=33.7\text{ ms},\;a_{\text{DA}}^{+}=1.3\times 0.03125,\;a_{\text{DA}}^{-}=-0.8\;\times \;0.85\;\times \;0.03125\right)$. Following learning, the weights were clipped to [0, 1].

**Figure 2:**
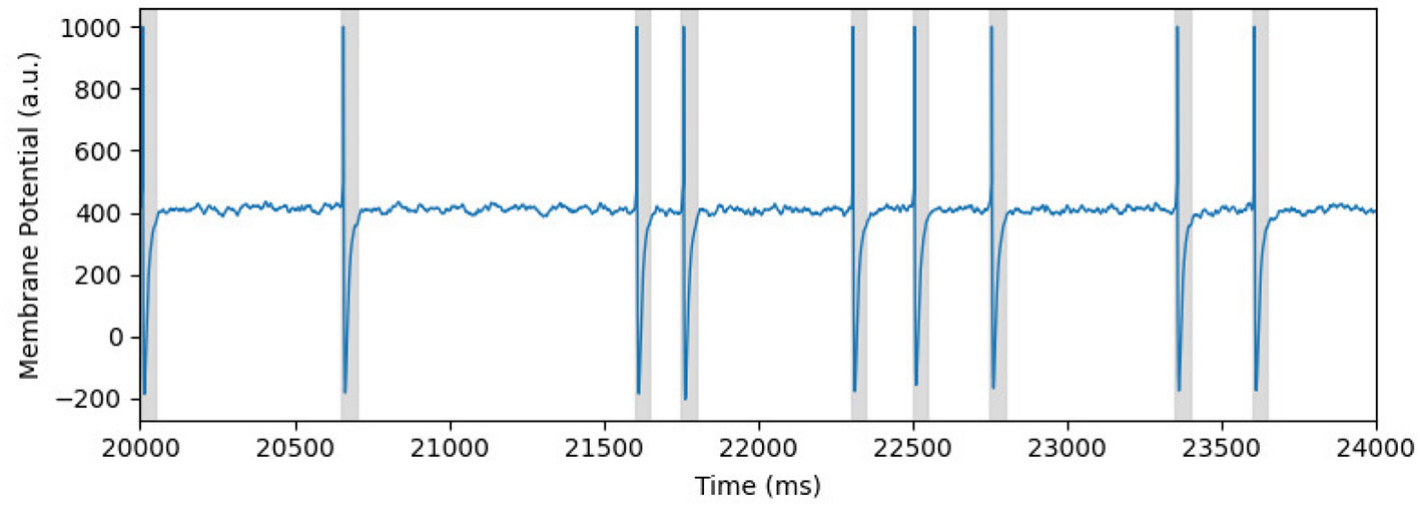
Example trace of the membrane potential of the leaky integrate-and-fire (LIF) neuron model. The gray boxes represent the 50 ms target patterns.

The LIF neuron eventually fires at an early timing in the target patterns after a learning period. Figure 2 shows a sample of the membrane potential trace.

### 2.2 Simulation setups

**Figure 3:**
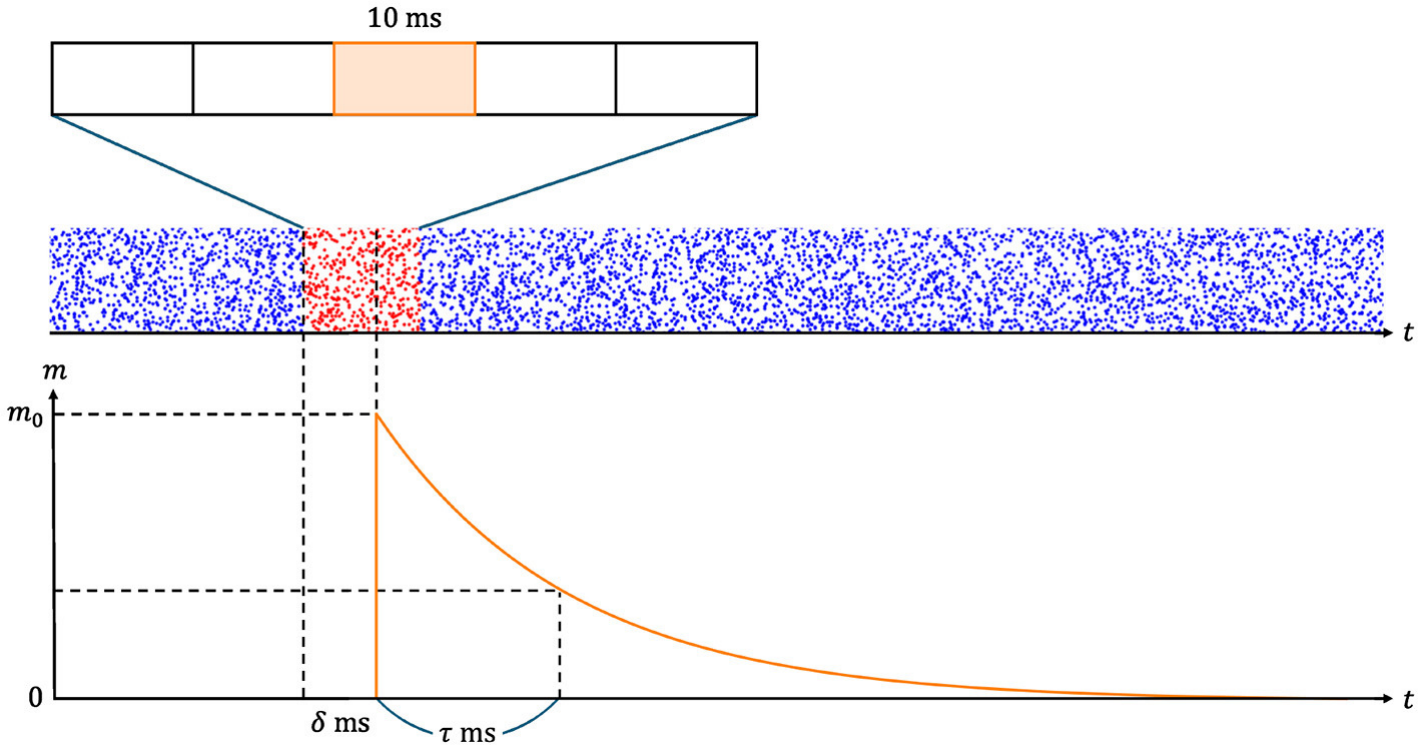
Graphical overview of our simulation setups. The upper part illustrates the (i) switched STDP curve model, where the spike-timing-dependent plasticity (STDP) curve is switched in one of the five slots (for example, the orange slot in the figure). The lower part illustrates the (ii) exponentially decaying DA model, where the dopamine (DA) decays after a certain delay.

Figure 3 illustrates a graphical overview of the setups.

#### 2.2.1 Switched STDP curve model

First, to investigate the effects of the DA-type STDP curve, we switched between the blue and orange curves in Figure 1.

The input spike trains were generated by the same procedure as in the study by Masquelier et al. (2008). Independent Poisson spike trains with rates 0–90 Hz for 2,000 synapses were generated. The first 50 ms spatio-temporal pattern was selected as the detection target, which was embedded with the rate of 10% (approximately twice per second). To reflect the spike timing fluctuation, a Gaussian jitter with a mean of 0 ms and standard deviation (SD) of 1–5 ms (1 ms step) was applied. Then, 10 Hz Poisson spikes were added to the spike trains.

The DA-type STDP curve was applied for all or a part of the 50 ms target spatio-temporal patterns. In case of the partial application, the detection target pattern (50 ms) was partitioned into five 10 ms slots (1–5). In a designated slot, the STDP curve was switched to the DA-type with a probability of 10%–100% in each simulation run. Because the effect of the DA-type STDP curve is very strong, as shown in the results section, we tested whether narrowing the time width and reducing the amplitude of the DA-type STDP curve could mitigate its effect. We uniformly scaled $\left(\tau_{\text{DA}}^{+},\tau_{\text{DA}}^{-}\right)$ by *r*_τ_∈{0.1, 0.2, …, 1.0} and $\left(a_{\text{DA}}^{+},a_{\text{DA}}^{-}\right)$ by *r*_*a*_∈{0.1, 0.2, …, 1.0}.

In addition to the DA application slot and the STDP curve scaling (*r*_τ_, *r*_*a*_), we examined the effects of the initial synaptic weight *w*_0_∈{0.30, 0.35, 0.40} and the simulation time *T*∈{50 s, 100 s, 200 s}. For each condition and each jitter level (1–5 ms), we simulated 50 independent spike trains with different random seeds (50 simulation runs for each parameter setting). A run was considered successful if, within the last 20 s, the detection rate was ≥ 98 % with zero false alarms.

#### 2.2.2 Exponentially decaying DA model

This setup aims to investigate the effect of DA in more realistic conditions. Here, we considered the dynamics of DA in brain tissue by incorporating its time constant. Considering that the time constant is about 400 ms (Beyene et al., 2019), the appearance rate of the target 50 ms pattern was reduced to 1% (approximately once per 5 s). In this setup, the spike-time jitter was fixed at 1 ms. The transmission delay of the DA pathway was also included in the model. Specifically, DA was assumed to be released with a fixed delay δ after the target pattern appeared, with a probability of 10%–100%. At the release of DA, the concentration *m* is reset to *m*_0_ and then decays exponentially according to the following equation.


$$\begin{array}{l} \frac{dm}{dt}=-\frac{m}{\tau_{\text{DA}}}. \end{array}$$ (7)

Here, δ∈{0 ms, 10 ms, 20 ms, 30 ms}, τ_DA_ = 400 ms, *m*_0_∈{0.01, 0.02, …, 0.05, 0.10, …, 0.50}. Note that *m* is an abstracted modulation variable rather than a direct measure of extracellular dopamine concentration or receptor occupancy. This linear scaling approximation was adopted to systematically explore the qualitative impact of modulation strength while keeping the model tractable. When *m* = 1.0, the STDP curve completely matches the DA-type one (the orange curve in Figure 1). The effective weight update under DA was a weighted average:


$$\begin{array}{l} \Delta w=\left(1-m\right)\Delta w_{\text{base}}+m\Delta w_{\text{DA}}r_{\text{DA}}, \end{array}$$ (8)

where Δ*w*_base_ and Δ*w*_DA_ are Δ*w* at the control condition and under DA, respectively. Parameter *r*_DA_ represents the efficacy of DA on the plasticity mechanism. A smaller value is selected for this scaling parameter to simulate a milder DA effect. We systematically varied *r*_DA_∈{0.1, 0.2, …, 1.0} and initial concentration *m*_0_∈{0.03, 0.05, 0.10, 0.15, …, 0.50} to examine its effect on synaptic modification. The simulation time was 200 s with an initial weight of *w*_0_ = 0.30. We used the last 50 s for the evaluation. The input spike trains and the detection success criterion were the same as the previous setup.

## 3 Results

### 3.1 Switched STDP curve model

We first examined the direct effect of the DA-type STDP curve on pattern detection using the switched STDP curve model.

For each parameter condition, we executed 50 simulation runs with independent Poisson input spike trains with different random seeds. For each trial, we evaluated the result using the last 20 s of the simulation data. A run was counted as successful when the detection rate in this evaluation window was at least 98% and no spike occurred outside the detection target patterns. Here, the detection rate is the rate of the bins of the detection target pattern with a spike divided by the total number of bins of the detection target pattern. In addition, we quantified the false alarm fraction, defined as the proportion of runs that satisfied the detection criterion (detection rate ≥98%) but produced at least one false alarm. Simulations were implemented in Brian 2 (Stimberg et al., 2019) using explicit Euler integration (Δ*t* = 0.1 ms). Random seeds were set as *S*_0_ = 1, 000 and *S*_*i*_ = *S*_0_+*i* (*i* = 0, …, 49).

When the DA-type STDP curve was applied all over the detection target pattern, the model never reached the success criterion: the success rate was 0% and all runs exhibited false alarms (100% false alarm indicating that uniform DA modulation makes the neuron excessively sensitive. Therefore, we applied DA-type STDP for only one 10 ms slot with a release probability (see Section 2.2.1).

**Figure 4:**
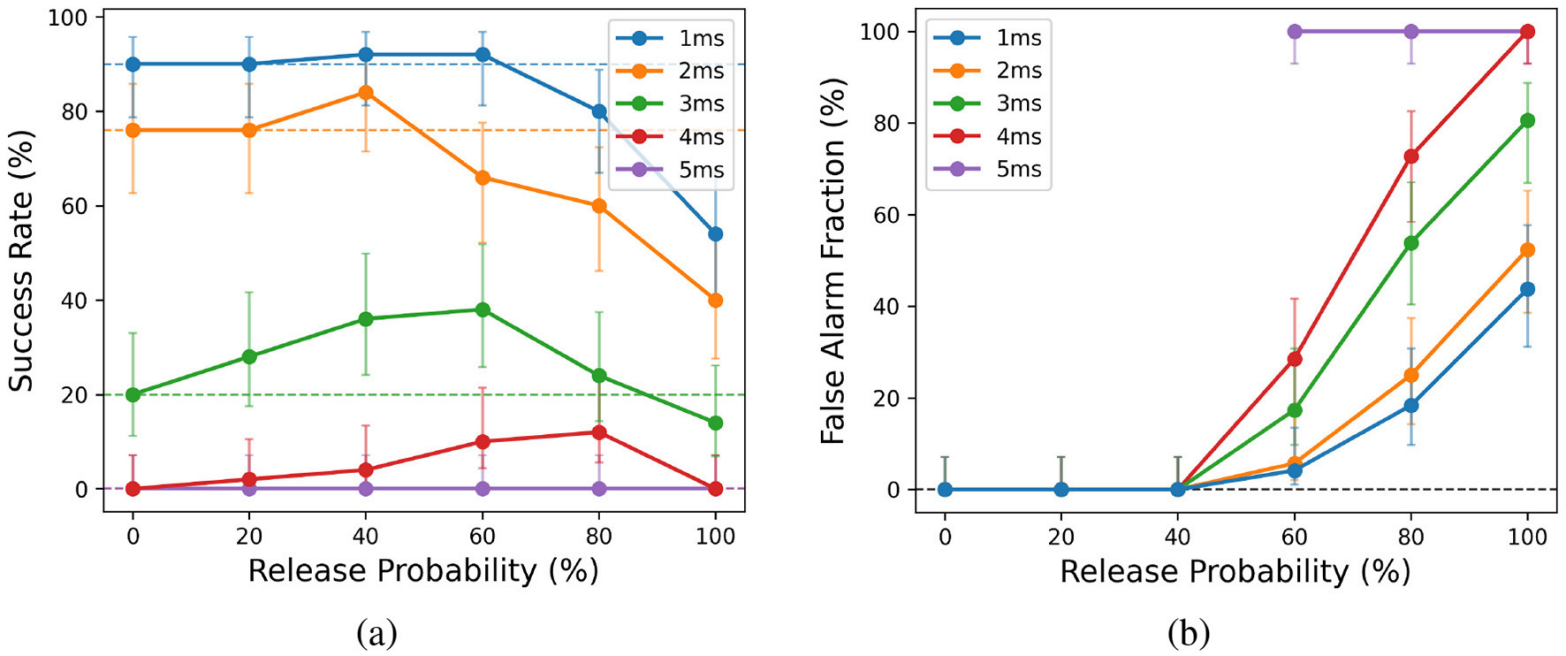
Dependence of **(a)** the detection success rate and **(b)** the false alarm fraction on dopamine (DA) release probability and spike-time jitter under the baseline condition: the initial synaptic weight was *w*_0_ = 0.30, *r*_τ_ = *r*_*a*_ = 1.0, DA was applied only in the third 10 ms slot of the 50 ms embedded pattern, and the total simulation time was 100 s. Each point represents the average over 50 runs, and the error bars indicate 95% Wilson confidence intervals for the binomial proportion (Newcombe–Wilson method). In **(b)**, some points are missing for 5 ms jitter because their success rate was zero.

Figure 4 shows the results with the baseline condition (*w*_0_ = 0.3, *r*_τ_ = *r*_*a*_ = 1.0, and T = 100 s). Each point represents the success rate, the proportion of successful runs among 50 runs. Here, error bars indicate 95% Wilson confidence intervals for the binomial proportion. When the jitter noise was moderate (2 ms), the moderate DA release probability (40%) improved the the detection success rate compared to the control condition without DA (the value at the release probability 0%). However, further increase of the release probability suppressed the success rate. This deterioration was accompanied by a marked increase in the false alarm fraction (see Figure 4b). The impact of DA was more pronounced for larger jitter values, but the success rate was lower across the release probability. While DA could enhance the success rate, it also facilitated potentiation for weakly related inputs and therefore made the neuron more prone to false detections, especially when the release probability was higher.

**Figure 5:**
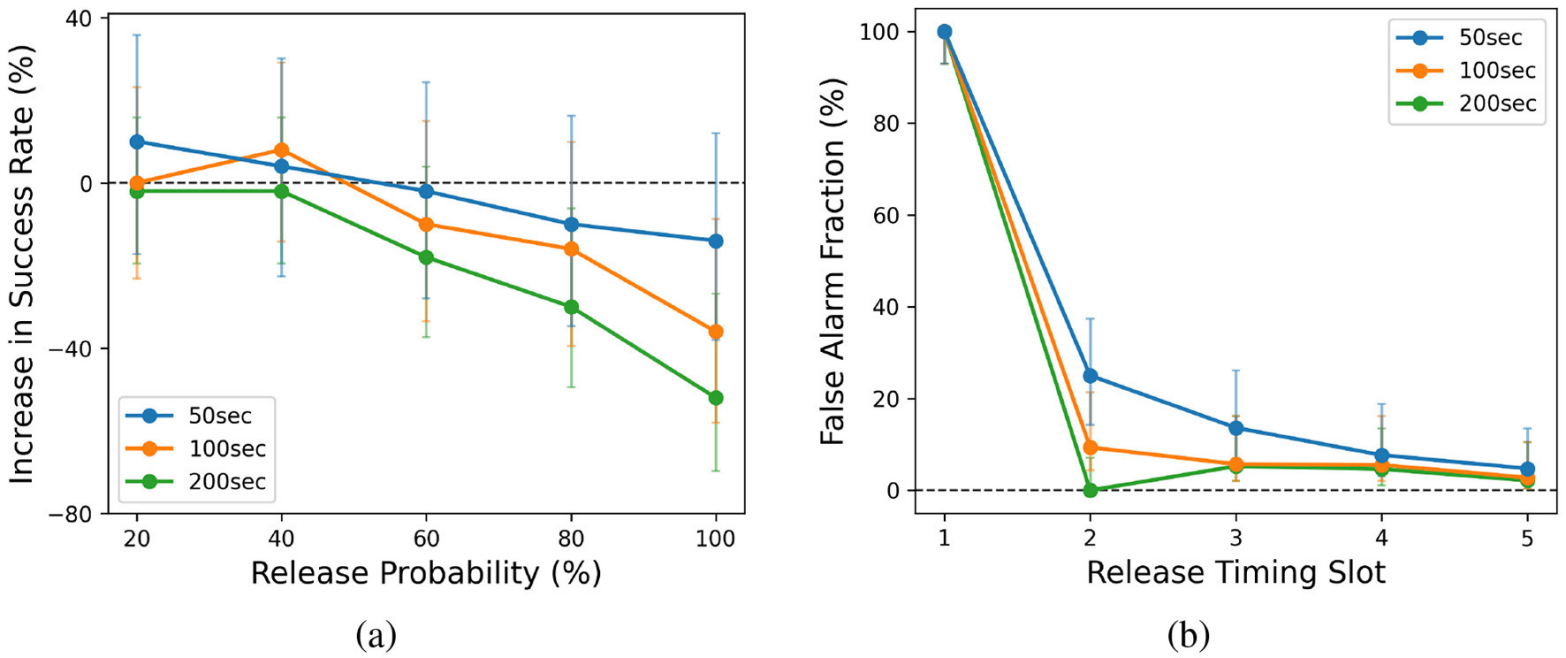
Dependence of the effect of dopamine (DA) on the learning duration *T* for jitter noise 2 ms (other parameters are as in Figure 4). **(a)** Increase in detection success rate relative to the control condition without DA; each point represents the average over *n* = 50 runs and the error bars denote 95% Wilson confidence intervals for the difference of binomial proportions (Newcombe–Wilson method). The line of 0% corresponds to the success rate for the release probability 0% for each value of *T*. **(b)** False alarm fraction as a function of the DA release slot (horizontal axis) at a release probability of 60%.

**Figure 6:**
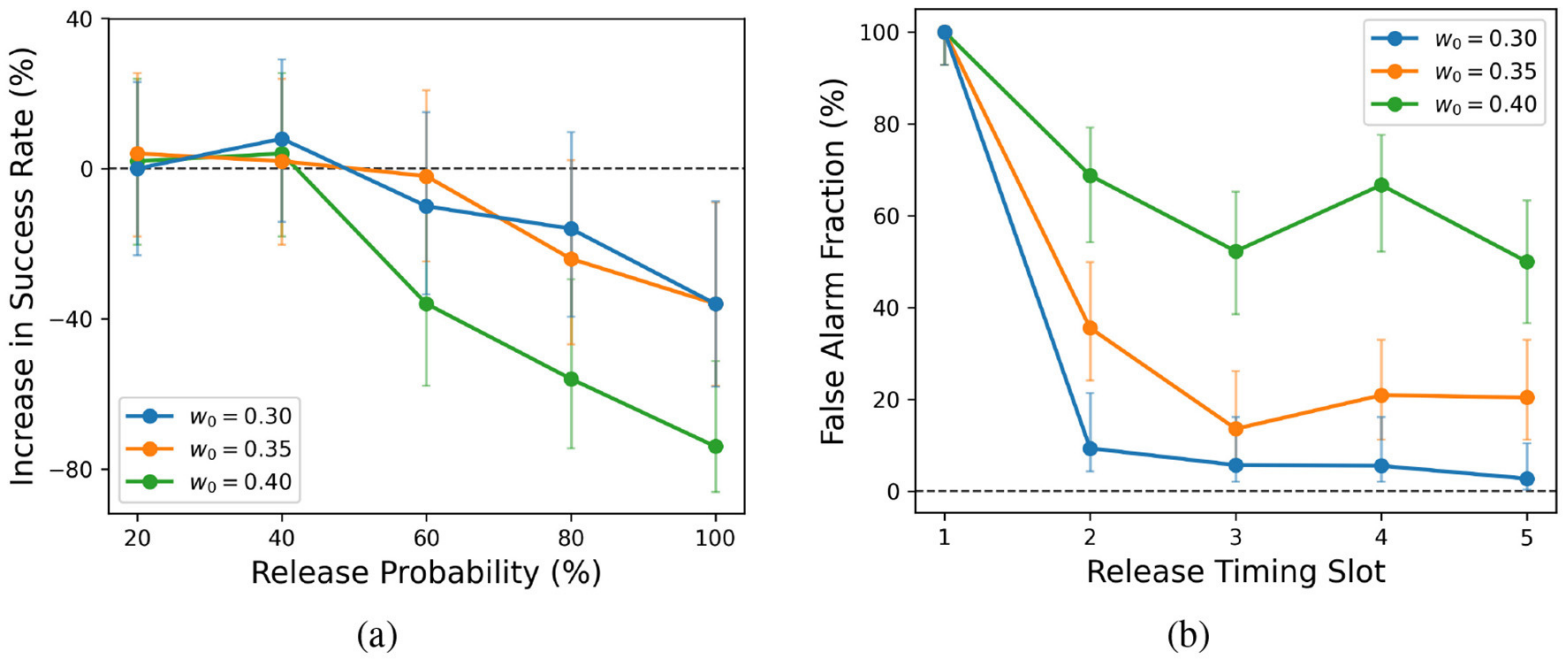
Dependence of **(a)** the success rate and **(b)** the false alarm fraction on dopamine (DA) on the initial synaptic wieght *w*_0_ for jitter 2 ms (other parameters are as in Figure 4). Each point represents the average over 50 runs, and the error bars indicate 95% Wilson confidence intervals for the binomial proportion (Newcombe–Wilson method).

To investigate the success rate enhancement effect for moderate jitter noise (2 ms), we first tested the dependence on the learning duration *T* (see Figure 5a). For a shorter learning duration (*T* = 50 s), moderate DA release (20%–40%) led to a clear gain in rate, whereas for a longer learning duration (*T* = 200 s) this benefit was reduced, especially when the release probability was higher, because incorrect synapses had more opportunities to be strengthened. These results suggest that the DA-type STDP curve helps quick learning by widening the Long-term potentiation (LTP) time window. Next, we visualized the dependence of the false alarm fraction on the release timing slot (Figure 5b). Here, the release probability was 60%. False alarms were particularly frequent when DA was released in the first slot of the pattern. In this case, DA was applied immediately after a random, non-pattern segment that would normally induce Long-term depression (LTD). The presence of DA converted these random presynaptic spikes into LTP, which reinforced irrelevant synapses. Finally, the dependence on the initial synaptic weight *w*_0_ was evaluated (Figure 6). Higher initial weights, corresponding to higher intrinsic excitability of the neurons, led to more frequent false alarms and diminished the beneficial effect of DA.

**Figure 7:**
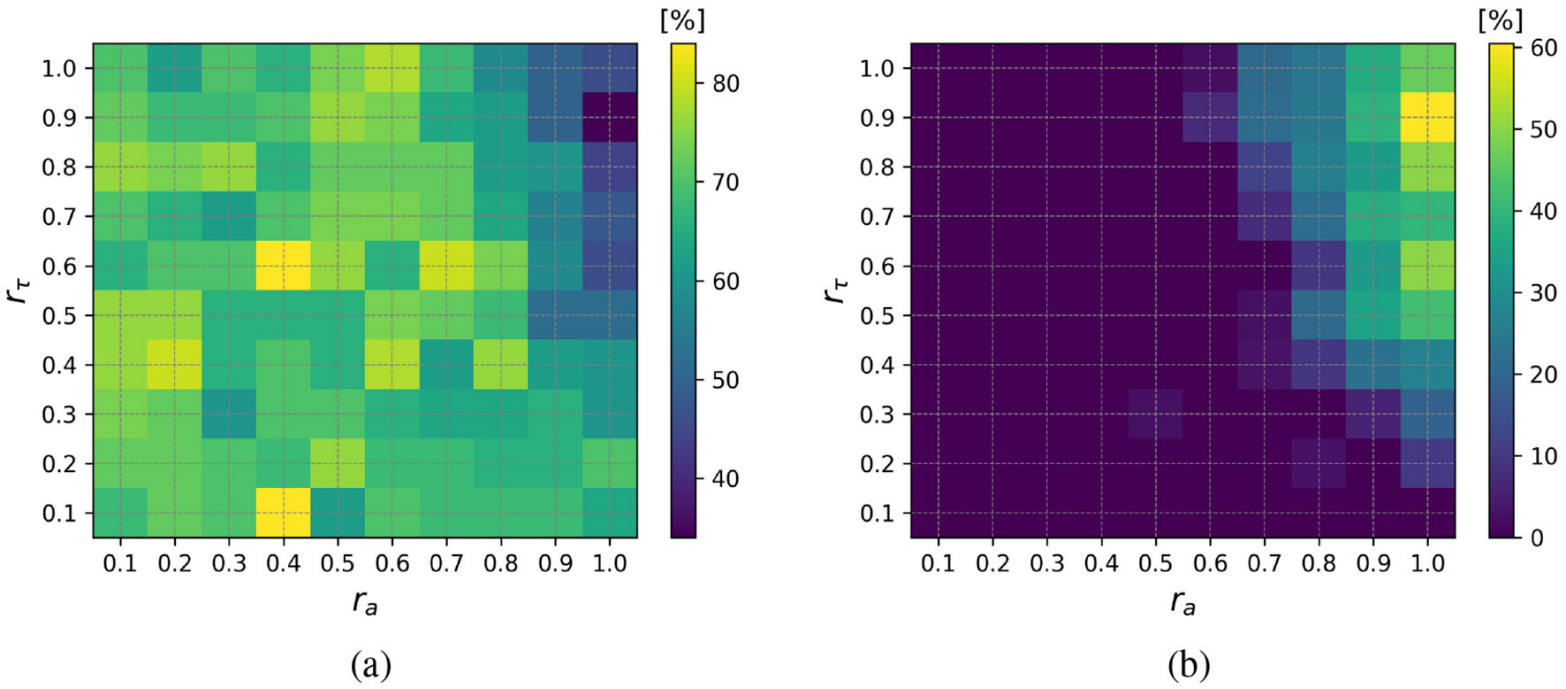
Effect of scaling the dopamine (DA)-type spike-timing-dependent plasticity (STDP) curve under the baseline condition and a DA release probability of 100%. **(a)** Detection success rate and **(b)** false alarm fraction as functions of the scaling parameters (*r*_*a*_, *r*_τ_). Each point indicates the average over 50 runs.

The above results indicate that the DA-type STDP curve has a trade-off between the beneficial (improving the success rate and accelerating the learning speed) and detrimental (increasing the false alarm fraction) effects. Here, we examined how this trade-off depends on the scale of the STDP curve. In this analysis, the time constant and amplitude of the DA-type STDP curve were multiplied by factors *r*_τ_ and *r*_*a*_, respectively. Figure 7 shows the dependence on (*r*_*a*_, *r*_τ_) for jitter 2 ms and DA release probability of 100%. Even at such a high release probability, narrowing the STDP window substantially suppressed the false alarm fraction. For instance, under the baseline condition (*r*_*a*_, *r*_τ_) = (1.0, 1.0) the detection success rate was only 46% and the false alarm fraction was comparably high (approximately 47%), whereas scaling the to (*r*_*a*_, *r*_τ_) = (0.4, 0.6) increased the success rate to 84% and eliminated false alarms (0%). The figures indicate that reducing *r*_*a*_ to half eliminates the false alarms and improves the success rate over 70%.

Overall, these results show that the DA-type STDP curve accelerates learning and enables rapid acquisition of the detection target pattern, but at the cost of “coarse” learning, the neuron tends to spike in response to non-target patterns. When the DA release probability or the baseline excitability is too high, the neuron is more likely to learn incorrect patterns and to fire in non-target patterns. By scaling the DA-type STDP curve, we could suppress these false alarms, even at high DA release probabilities, because this manipulation selectively weakens potentiation for non-matching inputs while preserving strong potentiation at the correct pattern timing.

### 3.2 Exponentially decaying DA model

Next, we examined how DA can modulate the spatio-temporal pattern-detection performance. As described in Section 2.2.2, the embedded pattern appeared with a probability of 1% and the DA concentration *m*(*t*) decayed exponentially in this model.

In the control condition without DA, the neuron detected only about 18% of the embedded patterns despite the long learning duration (*T* = 200 s).

**Figure 8:**
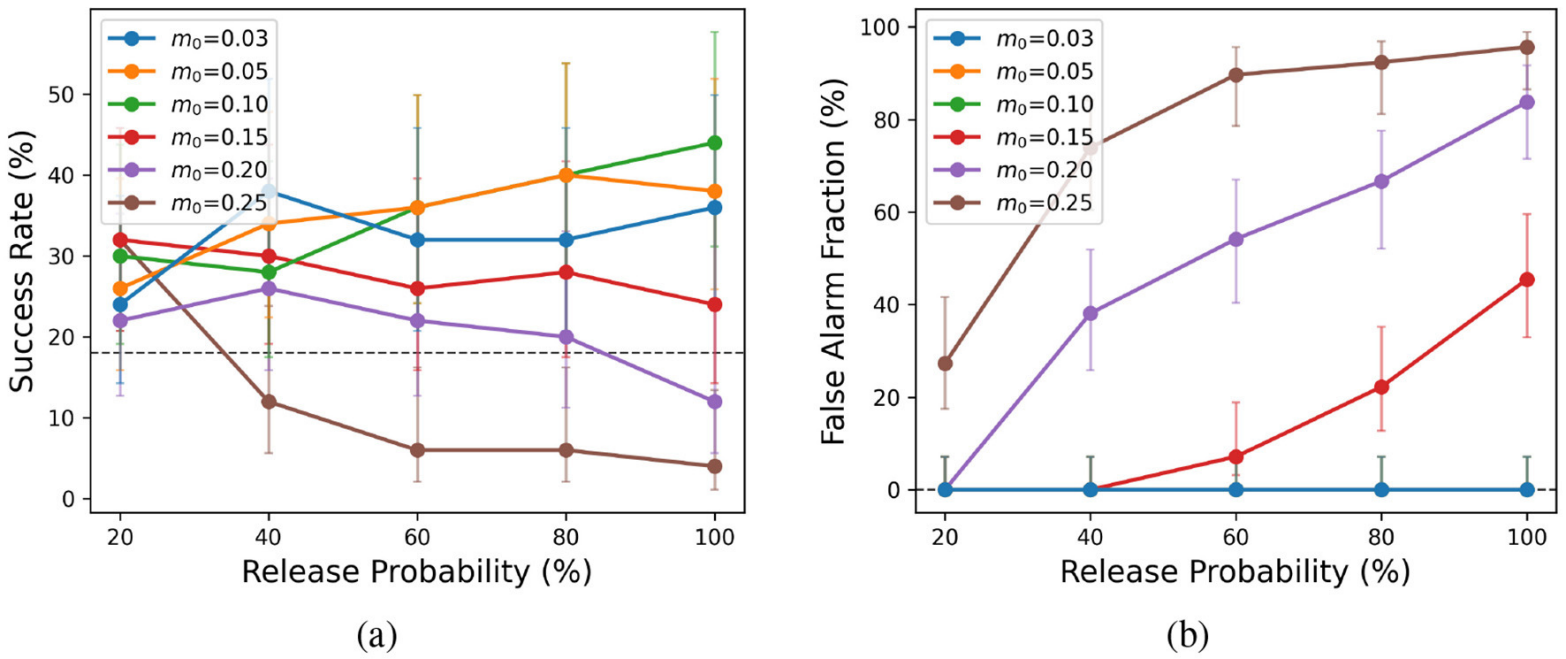
Dependence of **(a)** the detection success rate and **(b)** the false alarm fraction on initial dopamine (DA) concentration *m*_0_ and the DA release probability. Scaling parameter *r*_DA_ was fixed at 1.0 and delay δ was 10 ms. Each point represents the average over 50 runs, and the error bars indicate 95% Wilson confidence intervals for the binomial proportion (Newcombe–Wilson method).

To highlight the effect of DA dynamics, we first fixed the scaling parameter to *r*_DA_ = 1.0 and investigated how the initial concentration *m*_0_ and DA release probability affect the success rate and false alarm fraction (Figure 8). When *m*_0_ was larger than 0.10, the success rate tended to drop as the release probability increased (Figure 8a), which was accompanied by the increase of the false alarm fraction (Figure 8b). In contrast, when *m*_0_ was 0.10 or smaller, the success rate tended to increase in response to the increase in the release probability. When *m*_0_ was 0.10, the model yielded the best performance: the detection success rate reached 44% with no false alarms, 2.44 times higher than the 18% in the control condition without DA. Although a success rate of 44% at the single-neuron level may seem modest, it would provide a substantial gain when many neurons act as weak learners in parallel, suggesting that DA can enhance the ability to detect the events that occur less frequently in cortical circuits.

**Figure 9:**
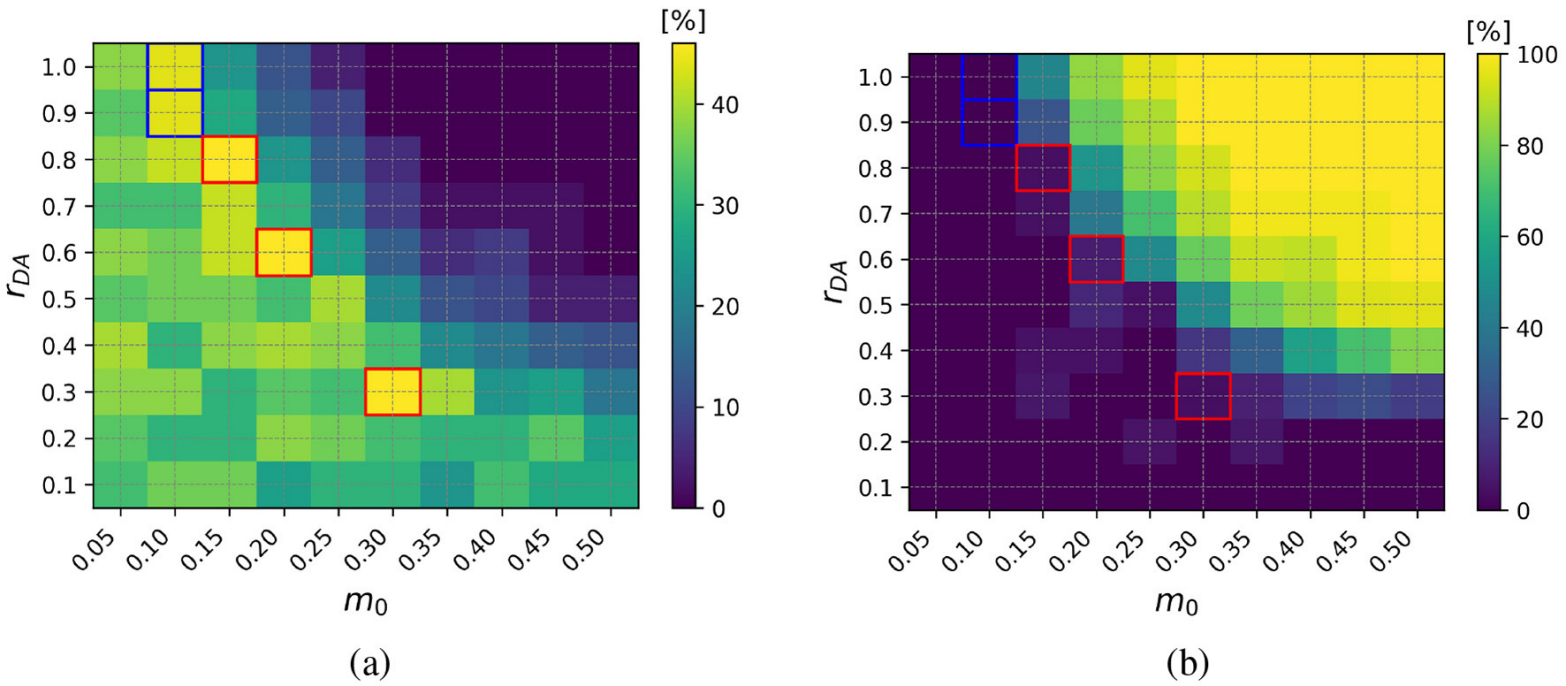
Dependence of the pattern-detection performance on initial dopamine (DA) concentration *m*_0_ and DA scaling parameter *r*_DA_. The panels show **(a)** the detection success rate and **(b)** the false alarm fraction as functions of *m*_0_ and *r*_DA_ for a fixed DA release delay δ. Delay δ was 10 ms and release probability was 100%. Each point indicates the average over 50 runs. In both panels, the points enclosed by a blue box indicate the best success rate with no false alarms. The points with a red box have a higher success rate, but with false alarms.

**Figure 10:**
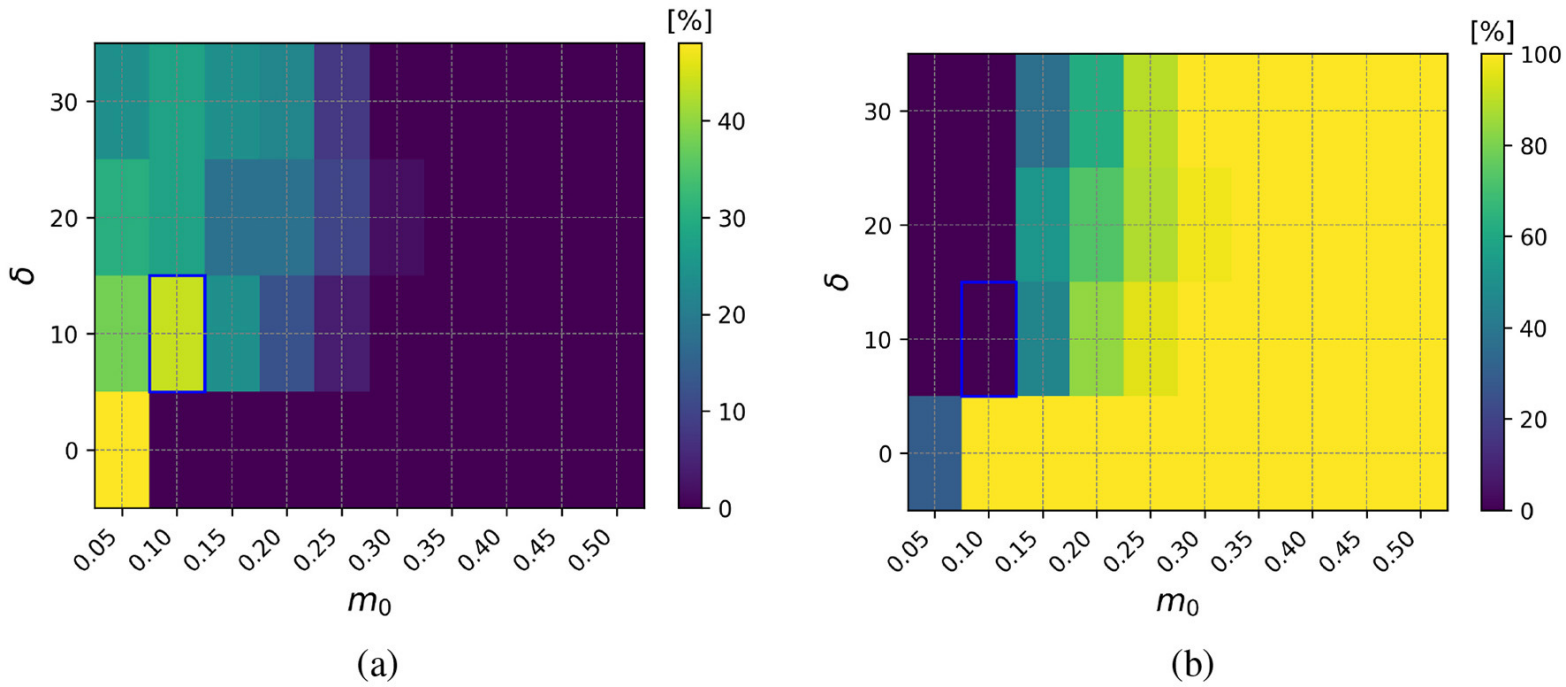
Dependence of the pattern-detection performance on initial dopamine (DA) concentration *m*_0_ and DA release delay δ. The panels show **(a)** the detection success rate and **(b)** the false alarm fraction. Scaling parameter *r*_DA_ was fixed at 1.0 and the release probability was 100%. Each point indicates the average over 50 runs. The point enclosed by a blue box indicates the best success rate with no false alarms.

Second, we examined how the DA scaling parameter *r*_DA_ and delay δ in the DA pathway influence the detection performance. Figure 9 shows how the detection performance depended on the combination of *r*_DA_ and *m*_0_. In the figure, δ was 10 ms. Weakening *r*_DA_ suppressed false alarms even when *m*_0_ was high. However, the best results were obtained when *m*_0_ was relatively low (0.10) and *r*_DA_ was high (0.9–1.0). The success rate was 44% without false alarms (the blue boxes in Figure 9a). When *r*_DA_ was lower, moderate *m*_0_ (0.3–0.8) yielded a higher success rate (48%) but with 4%–8% false alarm fraction (red boxes in the figure). In the figure, a high success rate tends to appear for larger *r*_DA_ when *m*_0_ is smaller. It is natural that there are best balance points between the initial concentration and the effectiveness of DA. However, the figure also indicates that a very low initial concentration yields relatively good performance regardless of *r*_DA_. Figure 10 shows the dependence on δ. When DA was released without delay (δ = 0 ms), the success rate was high only when *m*_0_ was very small, and was otherwise zero with frequent false alarms. Introducing a short delay of about δ = 10 ms markedly reduced the false alarm fraction while maintaining a high success rate, yielding the best overall performance (the blue boxes in Figure 10a). Further increasing the delay to δ≥20 ms suppressed false alarms more effectively, but the improvement in success rate remained limited. Thus, to maximize the success rate under the strict condition of zero false alarms, a relatively short delay of around 10 ms was optimal.

Overall, these results indicate that the exponentially decaying DA model also exhibits a trade-off between enhanced pattern detection and robustness against false alarms. The pattern-detection performance is governed by the interaction between initial DA level *m*_0_ and the scaling parameter *r*_DA_. The best success rate without false alarm was achieved when *m*_0_ was low and *r*_DA_ was high. But when *r*_DA_ was lower, moderate *m*_0_ could achieve good results. Larger *m*_0_ or higher *r*_DA_ increased the likelihood of false detections. Conversely, false alarms were suppressed when the DA effect was weak, even at high *m*_0_, but did not yield the best improvement in the success rate. Thus, an appropriate balance between the initial DA concentration and the “DA effect” represented by *r*_DA_ is suggested to be essential for reliable and accurate pattern detection. The importance of a short delay was also suggested.

### 3.3 An eligibility-trace-based model

The influence of dopamine on synaptic plasticity has also been studied in the context of reinforcement learning through the introduction of an eligibility trace. Here, we applied one of the models of Izhikevich (2007) to the spatio-temporal spike pattern detection task to see the effect of the eligibility trace compared to our model. In this model, a presynaptic spike induces the transient synaptic eligibility trace, and DA gates the conversion of this trace into an actual synaptic weight change. For the *j*-th synapse, the eligibility trace *c*_*j*_, synaptic weight *w*_*j*_, and DA concentration *m* are described as follows:


$$\begin{array}{ll} \frac{dc_{j}}{dt} & =-\frac{c_{j}}{\tau_{c}}+k_{w}\Delta w_{j}\left(\tau_{c}\right), \end{array}$$ (9)

$$\begin{array}{ll} \frac{dw_{j}}{dt} & =c_{j}m, \end{array}$$ (10)

$$\begin{array}{ll} \frac{dm}{dt} & =-\frac{m}{\tau_{m}}+k_{\text{DA}}DA\left(t\right), \end{array}$$ (11)

$$\begin{array}{ll} DA\left(t\right) & =0.01+0.5\delta \left(t-t_{\text{rel}}\right) \end{array}$$ (12)

where τ_*c*_ and τ_*m*_ are decay time constants and *k*_*w*_ and *k*_DA_ are scaling parameters. The values of the decay time constants are the same as those in Izhikevich (2007) (τ_*c*_ = 1 s and τ_*m*_ = 0.2 s). The scaling parameters *k*_*w*_ and *k*_DA_ roughly correspond to *r*_DA_ and *m*_0_ in our model, respectively. The former scales the amount of synaptic update, whereas the latter scales the amount of DA input. The DA input is expressed by *DA*(*t*), which is the same as that in Izhikevich (2007), where *t*_rel_ is the release time of DA. The scaling parameters were systematically varied *k*_*w*_∈{0.5, 1.0, …, 5.0} and *k*_DA_∈{0.5, 1.0, …, 5.0}. To make a fair comparison, *t*_rel_ was set identical to the DA input times in our model. Because DA signals are delivered with only a short delay in our model's setting, it departs from the classical reinforcement learning framework, in which DA is assumed to encode a reward prediction error delivered after a behavioral outcome.

**Figure 11:**
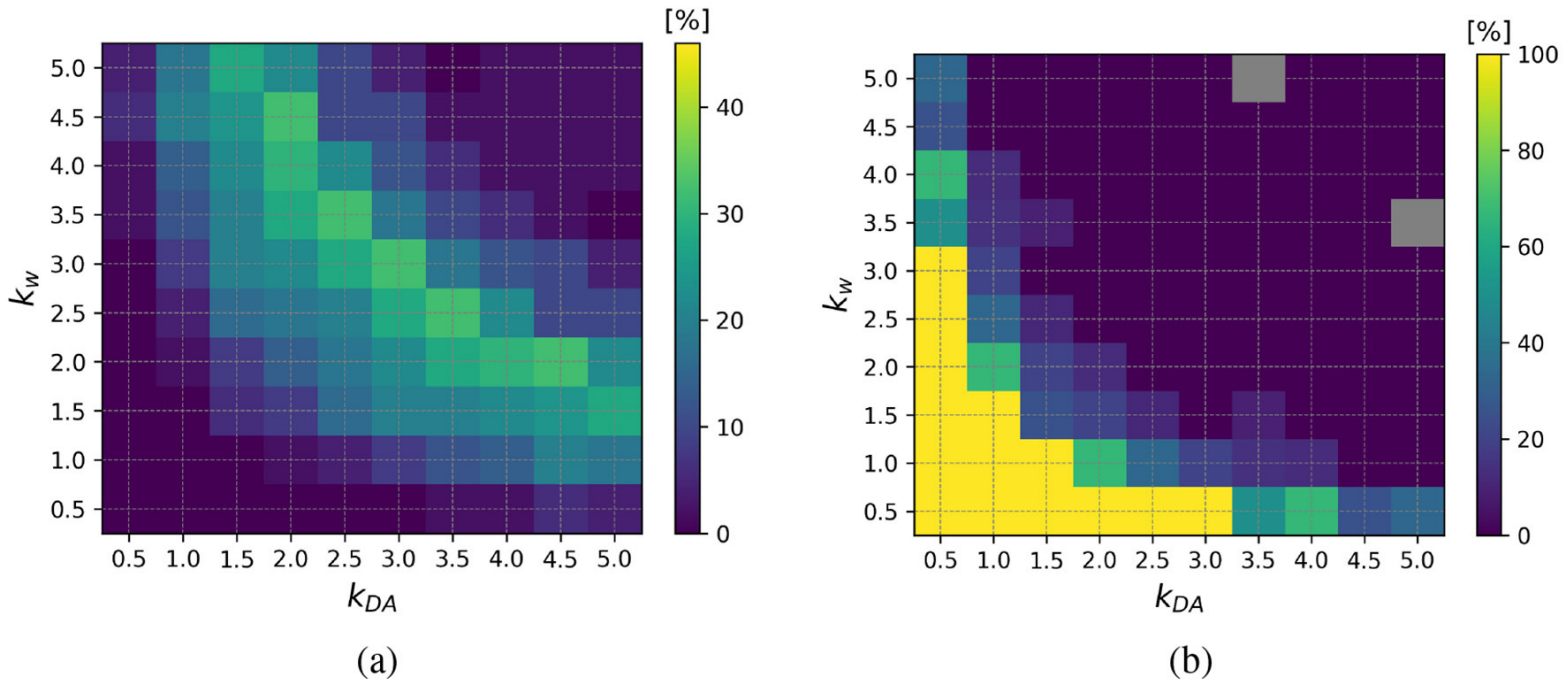
Dependence of the pattern-detection performance on scaling parameters for the spike-timing-dependent plasticity (STDP) amplitude and the dopamine (DA) concentration. **(a)** Detection success rate and **(b)** false alarm fraction as functions of the scaling parameters (*k*_*w*_, *k*_DA_). The gray boxes represent a missing value because of zero success rate. The simulation time was 500 s.

Figure 11 shows the dependence of the pattern detection performance on scaling parameters (*k*_*w*_, *k*_DA_) for this model. The simulation time was 500 s, which is 2.5 times longer than that for our model (200 s). It is because this model had a far poorer performance with the simulation time of 200 s. The tendency of the distribution of successful parameter combinations had a trade-off similar to that in our model (Figure 11a). However, the false alarm fraction had an opposite tendency (Figure 11b). The higher scaling parameters suppressed the false alarm rate. From the perspective of single-neuron information processing, directly modulating the STDP curve led to faster convergence than routing the weight change through an eligibility trace.

## 4 Discussion

In our model of Section 2.2.2, excessive DA led to an increase in false alarms, which degrades performance. By contrast, in the eligibility-trace-based model, the eligibility trace seems to act as a filter, suppressing false alarms by averaging out the transients. These results suggest that eligibility-trace-based STDP is effective as a synaptic learning rule even in a single-neuron context and outside the reinforcement learning framework, but that the inclusion of an eligibility trace introduces a fundamental speed-robustness trade-off. It confers greater resilience to noisy DA signals while substantially prolonging the time required for convergence.

Our finding that very high DA release probabilities degrade performance may appear to conflict with behavioral observations that learning tends to improve with a higher reward (Dickinson, 1994). One reconciling hypothesis is that the effective release probability of dopaminergic terminals is bounded below unity. The neurotransmitter release rate at synaptic terminals is inherently probabilistic and rarely approaches 100%. Under this view, the degraded-performance regime in our simulations may correspond to a non-physiological range rather than to conditions that would actually be realized by dopaminergic neurons *in vivo*. Indeed, several studies have probed such non-physiological regimes. Coddington and Dudman (2018) reported that driving midbrain dopamine neurons beyond physiological firing rates can elicit reward-learning-related movements, and Long et al. (2024) showed that elevated activity of dopaminergic neurons increases the information carried by striatal neurons. Our model may be expanded to explain these findings in the future.

In Figure 10, the release delay of about 10 ms induced the best overall performance. It is inconsistent with Yagishita et al. (2014) that reported the time delay of 0.6 s induced structural LTP dependent on dopamine in the Ventral Tegmental Area-Nucleus Accumbens (VTA-NAc) circuit most effectively. This may indicate that the plasticity in our model corresponds to functional STDP-based synaptic updates rather than structural LTP. The discrepancy is likely due to the distinction between the timescales of functional and structural plasticity, the former supports fast adaptation of neural activity to the environment, and the latter allows significant and long-duration changes (Bernardinelli et al., 2014). It might also be induced by the specific structure of the VTA-NAc circuit.

Although this work aims a general model, the model was constructed based on hippocampal data from Zhang et al. (2009), in which dopamine was applied exogenously to cultured hippocampal neurons. Thus the model may inherit properties specific to dopaminergic modulation of hippocampal synapses. Therefore, validation is required when this model is applied to other brain regions.

## 5 Conclusion

In this study, we conducted a bottom-up analysis to clarify how DA affects neural computation at the circuit level by focusing on a single neuron, the most fundamental unit of neural circuits. By incorporating a DA input into the spatio-temporal spike pattern detection model of Masquelier et al. (2008) and modeling DA effects through the DA-type STDP learning rule reported by Zhang et al. (2009), we investigated the effect of DA on a single neuron's information processing.

Across two simulation setups, we consistently found that DA modulation introduces a trade-off between sensitivity (rapid learning and higher detection success) and specificity (robustness against false alarms). In the switched curve setup, the DA-type STDP curve was shown to accelerate learning and enable rapid acquisition of the target pattern, but also had a tendency to promote coarse learning that increased the number of false alarms. In the exponentially decaying DA setup, performance depended on the interaction among the initial DA concentration *m*_0_, the DA scaling parameter *r*_DA_, and the release delay δ. It was suggested that a lower initial DA concentration and a DA release delay of 10 ms or longer are advantageous for spatio-temporal spike pattern detection capability. The success rate reached 44% (2.44 times the control condition without DA) with zero false alarms, indicating that DA can substantially enhance detection of infrequent events even at the single-neuron level when its magnitude and timing are appropriately balanced. It was also suggested that suppressing the initial DA concentration can effectively eliminate the false alarms without critically affecting the success rate.

These findings provide a bottom-up perspective: dopaminergic modulation can maintain good learning and detection of behaviorally relevant patterns, but excessive DA effect or mistimed DA signals may increase false detections by reinforcing non-target inputs. This sensitivity–specificity trade-off suggests that not only the amount of DA, but also its temporal alignment with synaptic events is critical for reliable information processing through plasticity. The relative insensitivity of the success rate to DA concentration compared to false detection suggests that suppressing the amount of DA release may be a good strategy to suppress false detection without critically damaging learning ability.

Future work includes extending to multiple neurons and developing specific models that consider the midbrain dopaminergic input and effects of other neuromodulators such as noradrenaline in hippocampal CA1, CA3, and the dentate gyrus (Kobayashi et al., 2022). Our model adopted a simplified dynamics for the receptor binding kinetics (Equation 7). However, considering biologically realistic dynamics, including receptor subtype (D1- and D2-like), is a crucial direction to strengthen the biological plausibility. These extensions may help bridge circuit-level principles with the cognitive impairments associated with neuropsychiatric disorders.

## Data Availability

The raw data supporting the conclusions of this article will be made available by the authors, without undue reservation.
